# Genealogical typing of *Neisseria meningitidis*

**DOI:** 10.1099/mic.0.031534-0

**Published:** 2009-10

**Authors:** Xavier Didelot, Rachel Urwin, Martin C. J. Maiden, Daniel Falush

**Affiliations:** 1 Department of Statistics, University of Warwick, UK; 2 Department of Biology, Pennsylvania State University, USA; 3 Department of Zoology, University of Oxford, UK; 4 Environmental Research Institute, University College Cork, Ireland

## Abstract

Despite the increasing popularity of multilocus sequence typing (MLST), the most appropriate method for characterizing bacterial variation and facilitating epidemiological investigations remains a matter of debate. Here, we propose that different typing schemes should be compared on the basis of their power to infer clonal relationships and investigate the utility of sequence data for genealogical reconstruction by exploiting new statistical tools and data from 20 housekeeping loci for 93 isolates of the bacterial pathogen *Neisseria meningitidis*. Our analysis demonstrated that all but one of the hyperinvasive isolates established by multilocus enzyme electrophoresis and MLST were grouped into one of six genealogical lineages, each of which contained substantial variation. Due to the confounding effect of recombination, evolutionary relationships among these lineages remained unclear, even using 20 loci. Analyses of the seven loci in the standard MLST scheme using the same methods reproduced this classification, but were unable to support finer inferences concerning the relationships between the members within each complex.

## INTRODUCTION

The demonstration that some bacterial pathogen populations are structured into lineages that differ in their propensity to cause disease represents a major achievement of molecular epidemiology (Musser, 1996). A striking example is *Neisseria meningitidis*, the meningococcus, which is a commensal inhabitant of the human nasopharynx that occasionally invades the host, causing devastating septicaemia and meningitis (Stephens, 2009). Whereas asymptomatic carriage of meningococci is common, the incidence of meningococcal disease is low, and most cases are caused by a limited number of genetic types known as the hyperinvasive lineages (Caugant, 2008; Yazdankhah *et al.*, 2004). There are several practical applications of these observations. Public health interventions can be targeted at particular lineages, as achieved with strain-specific meningococcal vaccines deployed in several countries (Jodar *et al.*, 2002). Furthermore, such interventions should avoid changing the ecology of the meningococcus in ways that might favour the spread of vaccine escape variants or more invasive genotypes that would increase the overall disease burden (Maiden & Spratt, 1999). Finally, this population structuring enables genetic differences between invasive and non-invasive meningococci to be defined, helping to elucidate mechanisms of pathogenicity (Bille *et al.*, 2008, 2005; Hotopp *et al.*, 2006).

Populations of bacteria contain identifiable phenotypic lineages due to clonal descent (Levin, 1981). Horizontal genetic exchange via homologous recombination reassorts variation progressively, so that isolates sharing a sufficiently recent common ancestor share a majority of genetic traits, including those that govern pathogenicity. In the case of the meningococcus, disease-associated, or ‘hyperinvasive’, lineages are apparent from the characterization of isolate collections by multilocus enzyme electrophoresis (MLEE), multilocus sequence typing (MLST) and, to an extent, serological isolate characterization (Caugant *et al.*, 1987; Maiden *et al.*, 1998; Urwin *et al.*, 2004). The principal aim of any microbial typing methodology is to find, within a sample of isolates, groups that share a recent common ancestor (van Belkum *et al.*, 2001). If all such clonal groups were known, along with an estimate of the relative times at which they shared common ancestry, then this knowledge could be represented as a tree called the clonal genealogy (Guttman, 1997). Reconstructing a clonal group of isolates is made possible by identifying the genetic modifications that occurred on the branch of the clonal genealogy directly above the group, which are likely to differentiate the members of the group from the rest of the isolates. However, two difficulties may occur when attempting to reconstruct clonal groups: first, there may be little genetic difference among the members of a group and their closest relatives, especially for recent groups (Achtman, 1996); and second, horizontal genetic exchange with related bacteria outside the group may obscure the signal of clonal inheritance, especially for older groups (Holmes *et al.*, 1999; Schierup & Hein, 2000).

The level of genotypic characterization necessary for a given isolate collection depends on the goals of the study (Struelens, 1998) and how often the phenotypes of interest, e.g. antigenic variants or pathogenicity, change. Since recombination reduces phenotypic correlations among related isolates, the inability to resolve the deep phylogeny does not affect many epidemiological analyses. Conversely, closely related isolates are likely to be phenotypically uniform, unless there is strong natural selection favouring repeated changes of particular traits. Therefore, fine-resolution dissection of these relationships is unlikely in any particular instance to provide substantial insight into the relationship between phenotype and genotype. It is, however, necessary to resolve these parts of the genealogy, for example to investigate recent outbreaks (Bygraves *et al.*, 1999; Feavers *et al.*, 1999).

In this study, an extended meningococcal MLST dataset comprising nucleotide sequence data from 20 housekeeping loci was analysed with the model-based method ClonalFrame (Didelot & Falush, 2007), which attempts to reconstruct the clonal genealogy while taking into account the statistical uncertainty of individual groups and the confounding effect of recombination. This analysis enabled an assessment of the accuracy and limitations of the MLST scheme in terms of the number of evolutionary relationships that it can correctly infer. The data show that the widely applied seven-locus MLST approach successfully identifies relationships over a narrow age range, corresponding to previously described clonal complexes, but has no power to detect longer-term relationships among these complexes. The resolution of closely related meningococcal isolates by seven-locus MLST can be poor and may need to be supplemented with additional information in certain circumstances.

## METHODS

### Sequence data.

The 107 isolates used to develop the *N. meningitidis* MLST scheme were investigated (Maiden *et al.*, 1998). This collection was designed to represent the global diversity of meningococcal disease in the latter half of the 20th century, and included multiple representatives of the hyperinvasive lineages identified at the time the collection was assembled. For a subset of 95 isolates, chosen to include all of the representatives of the major hyperinvasive lineages, published data from 12 gene fragments (Holmes *et al.*, 1999; Maiden *et al.*, 1998) were analysed together with data from an additional eight loci (Supplementary Table S1, available with the online version of this paper), giving a total of 20 loci. The additional loci were all annotated as encoding enzymes of cellular metabolism: *aspA* (aspartate ammonia-lyase); *carB* (carbamoyl phosphate synthase large subunit); *dhpS* (dihydropteroate synthase); *glnA* (glutamine synthetase); *gpm* (phosphoglycerate mutase); *pyk* (pyruvate kinase); *rpiA* (ribose-5-phosphate isomerase A); and *tal* (transaldolase) (Table 1). With the exception of the *glnA*, *aroE* and *mtg* loci, which were all located within a region of the genome 8.8 kbp in size, the 20 loci were distributed around the meningoccal FAM 18 genome (Bentley *et al.*, 2007) and were at least 27 kbp from each other (Supplementary Table S2). With the exception of the three loci mentioned, they were therefore highly unlikely to be co-inherited in a single transformation event, as the average recombination fragment size in meningococci has been estimated as between 1.1 kbp (Jolley *et al.*, 2005) and 5 kbp (Linz *et al.*, 2000).

Target gene fragments were amplified by the PCR and sequenced using specific oligonucleotide primers. The primers used for the *aspA*, *carB*, *dhpS* and *glnA* gene fragments have been described previously (Tondella *et al.*, 1999). The primers used for the remaining gene fragments were as follows: *rpiA*, 5′-CGA CAC AAG ACG AAC TCA AGC GC-3′ and 5′-GGC AGG GAT AGA TGA CTT TCG C-3′; *tal*, 5′-ATC GGA CGT TAA AGC ATT AGG-3′ and 5′-TTA AAC CAA CGG TGC GAG CAG-3′; *gpm*, 5′-CCA CTT TCA CAC ATT GGA GAC G-3′ and 5′-CGA TGA CTT TCA ATT GTC GTC C-3′; *pgk*, 5′-TTA TTT GAC GCG CAG CAC TTC C-3′ and 5′-TTA TTT GAC GCG CAG CAC TTC C-3′. Nucleotide sequencing was done as described previously (Jolley *et al.*, 2000). Briefly, genes were amplified by the PCR and purified using PEG_8000_/NaCl precipitation. The purified amplicons were used in extension reactions with ABI BigDye dye terminators, precipitated and separated on an ABI 3730 DNA Analyser. The sequences were assembled with the Staden suite of computer software (Staden, 1996) and aligned using the GCG package (Womble, 2000). The sequences have been deposited in the *Neisseria* Multi Locus Sequence Typing Database at http://pubmlst.org/neisseria/ (Jolley *et al.*, 2004).

### Inference of clonal relationships.

The principal analysis tool was ClonalFrame version 1.1 (Didelot & Falush, 2007), a statistical algorithm that infers clonal relationships while taking into account the effect of homologous recombination. ClonalFrame draws inference using a Monte-Carlo Markov chain, and therefore requires an assessment of the convergence and mixing of its results. Performing 10 independent runs of ClonalFrame of 200 000 iterations each achieved this. The first half of each run was discarded as burn-in, and the parameters were recorded every 200 iterations in the second half to produce samples of size 500 from the posterior distribution of clonal genealogies. The results from the 10 runs were compared for convergence by manual examination of the trace of the parameters and likelihood (Gelman, 1996), using the Gelman and Rubin statistic (Brooks & Gelman, 1998; Gelman & Rubin, 1992), and using the ClonalFrame genealogy comparison tool (Didelot & Falush, 2007). The convergence was judged satisfactory in all cases, and the samples from the 10 runs were combined to achieve maximum robustness. Statistical support for any grouping of isolates was assessed by the proportion of clonal genealogies exhibiting this grouping in the combined sample. This approach was independently applied to the extended (all 20 loci) dataset and to the MLST dataset (i.e. the seven fragments from genes *abcZ*, *adk*, *aroE*, *fumC*, *gdh*, *pdhC* and *pgm*). Missing data, namely the sequences of the eight novel gene fragments for 14 isolates as well as that of *gpm* for isolate BZ 147, were represented by a string of Ns in the ClonalFrame input file. The values of *r*/*m* (the ratio of rates at which a given nucleotide becomes substituted through recombination and mutation) and *ρ*/θ (the ratio of rates at which recombination and mutation events occur), as well as the relative age of the groupings, were estimated using ClonalFrame.

### Comparison of genealogies inferred with seven and 20 loci.

The quality of the genealogy reconstructed on the basis of the seven MLST loci (corresponding to ∼1.5 % of the entire genome of *N. meningitidis*) was assessed by comparison with the reconstruction based on the nucleotide sequences from all 20 loci (∼4.3 % of the entire genome). Assuming the genealogy reconstructed using the 20 loci to be predominantly correct, this gave a measure of inferential power of the seven-locus dataset. We also considered the support given by the 20-locus analysis to the clusters found by the seven-locus analysis. Under the same assumption, this gave a measure of the accuracy of the inference based on the seven-locus (i.e. MLST) dataset. Taken together, these two measures, power and accuracy, revealed the quality of the genealogical reconstruction based on the seven MLST gene fragments.

## RESULTS AND DISCUSSION

### Novel nucleotide sequences

Complete sequences for the gene fragments at the eight additional loci were obtained from 92 of the 95 isolates examined. Isolates 371 (ST-1 complex) and N45/96 (ST-41/44 complex) failed at all loci for technical reasons and no sequence was obtained for the *gpm* locus of BZ 147, possibly due to the absence of this gene in this isolate. The sequence gene fragments ranged in size from 366 bp (*dhps*) to 561 bp (*rpi*) in length with between 11 (*aspA* and *glnA*) and 33 (*dhps*) alleles per locus in the 93 isolates examined (Table 1). Of the new loci, the least diverse was *gpm* (5 % polymorphic sites) and the most diverse was *dhps* (21.2 % polymorphic sites); alternations in this gene confer resistance to sulphonamides (Kristiansen *et al.*, 1990). All novel gene fragments, including *dhps*, were subject to stabilizing selection, with *d*_N_/*d*_S_ ratios ranging from 0.071 (*pyk*) to 0.273 (*tal*). Allele numbers were assigned to each unique gene sequence and the data are available at http://pubmlst.org/neisseria (Table 1, Supplementary Table S1).

### Genealogical inference

**Fig. 1. f1:**
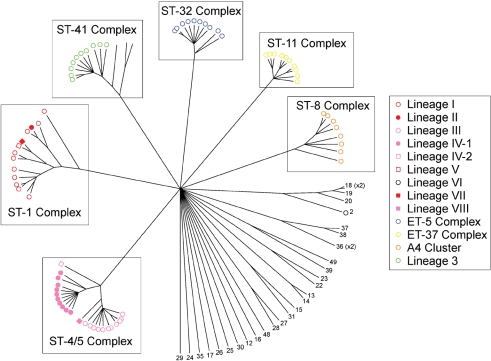
Majority-rule consensus tree for the clonal genealogies inferred by ClonalFrame when using all 20 gene fragments. The isolates are represented according to their MLEE designations, as shown in the key, and with no symbol meaning that no MLEE designation corresponds to the electrophoresis type of that isolate. ST numbers are shown for the isolates that do not belong to any of the six hyperinvasive complexes; see Fig. 3 for the others.

The genealogy inferred by ClonalFrame using 20 gene fragments clustered 78 of the 107 isolates into six lineages (Fig. 1). These corresponded to the major hyperinvasive groups identified by MLST and MLEE analyses of the same isolate collection (Achtman, 1994; Maiden *et al.*, 1998). For ease of comparison with other analyses, the ClonalFrame lineages were named according to the seven-locus MLST sequence type (ST) that they predominantly contained, which replicated MLST clonal complex designations. All of the isolates previously thought to belong to hyperinvasive lineages were located in one of these lineages, with the exception of isolate 322/85 (ST-2), which was characterized as lineage VI by MLEE (Fig. 1).

ClonalFrame grouped the MLEE serogroup A subgroups I, II, V and VII into the ST-1 complex, together with an additional serogroup B strain isolated in the Netherlands in 1977. On the basis of this analysis, MLEE subgroup I should not be differentiated from subgroups II, V and VII (Wang *et al.*, 1992). No relationships were detected between ST-1 complex and the remaining serogroup A isolates (designated subgroups III, VII, IV-1 and IV-2 by MLEE and ST-4 and ST-5 complexes by MLST), which were clustered into a single lineage with two major branches, referred to here as the ST-4/5 complex. MLEE gave an identical branching order for these subgroups (Achtman, 1994). The ST-41 isolates were closely related to those with ST-44; this close relationship was consistent with the MLST designation of the ST-41/44 complex, which contains both these STs. ClonalFrame could not reconstruct the relationship of the six lineages with each other and with the remaining isolates in the collection. Among these, few phylogenetic relationships were found, reflecting their high overall genetic diversity as compared to the isolates within each of the six lineages.

**Fig. 2. f2:**
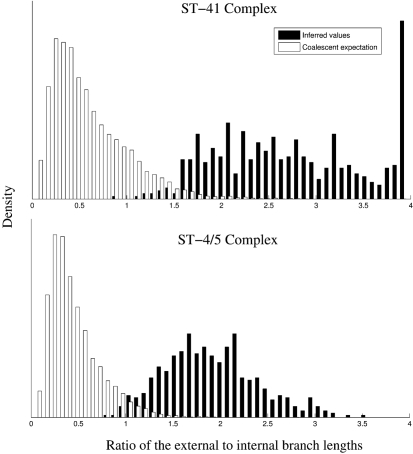
Inferred (black) and expected (white) values for the ratio of the external to internal branch lengths in the ST-4/5 complex and in the ST-41 complex. Expectations were estimated under the neutral coalescent model (Kingman, 1982a, b).

The credibility intervals for the relative ages of the six hyperinvasive lineages all included the value 0.2, indicating that they were of a similar age and that none represented a markedly recent epidemic clone (Smith *et al.*, 1993) (Table 2). Since the foundation of each hyperinvasive lineage, the members had mutated at up to 0.14 % of nucleotides, and 7–38 % of the genes analysed had been affected by homologous recombination, resulting in up to 1.5 % of nucleotides being substituted (Table 2). Although none of the complexes were young, two showed evidence for rapid expansion (Fig. 2), indicated by a higher ratio of external to internal branch lengths (Fiala & Sokal, 1985) than expected under the neutral coalescent model (Kingman, 1982a). This observation was consistent with these two lineages undergoing rapid population expansion early in their history, perhaps due to a fitness advantage, and explained the high number of polymorphic sites and polymorphic fragments observed in these two complexes. The branching patterns observed in the other four complexes showed some evidence of divergence from coalescent expectation too, but not at a statistically significantly level (Table 2).

### Effect of recombination

Nucleotide sequence data gathered by MLST can be used to estimate the relative importance of recombination and mutation. A parameter that is often used for this purpose is *ρ*/θ, the ratio of rates at which recombination and mutation events occur (Milkman & Bridges, 1990). The estimates for *ρ*/θ computed here were similar from one lineage to another, ranging from 1.5 to 7.7 (Table 2), indicating that recombination occurred up to eight times as often as point mutation in the evolution of *N. meningitidis* lineages. This was consistent with the estimates found by a number of previous studies: 3.6 (Feil *et al.*, 1999), 4.75 (Feil *et al.*, 2001), 1.1 (Fraser *et al.*, 2005) and between 0.16 and 1.83 (Jolley *et al.*, 2005). However, since recombination is likely to substitute more than one nucleotide at a time, the net effect of recombination over mutation is greater than this. This can be measured using *r*/*m*, the ratio of rates at which a given nucleotide becomes substituted through recombination and mutation (Guttman & Dykhuizen, 1994). Our estimates of *r*/*m* ranged from 14.4 to 108.9 (Table 2). This was slightly lower than previous estimates of *r*/*m* based on counting techniques, which were between 100 and 275 (Feil *et al.*, 1999, 2001; Jolley *et al.*, 2000), but not as low as a previous population genetics estimate, which was between 6.2 and 16.8 (Jolley *et al.*, 2005). The variation of *r*/*m* observed across lineages might account for part of the differences between previously reported values since these were based on datasets with different sampling frames.

**Fig. 3. f3:**
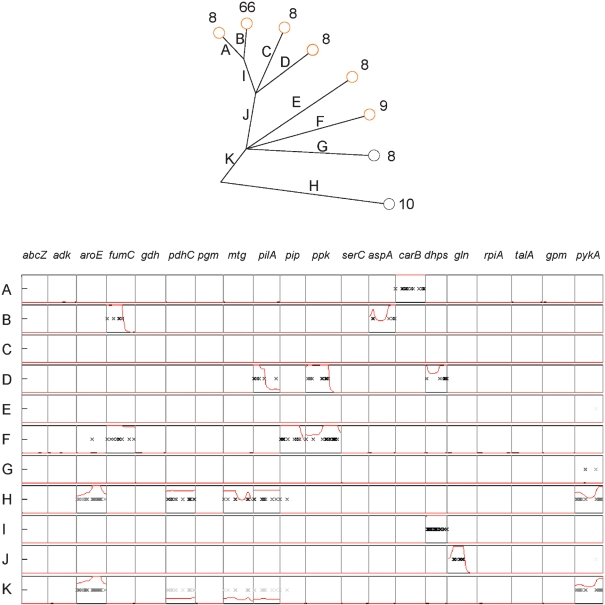
Evolutionary events inferred by ClonalFrame on the branches of the ST-8 complex for the 20-locus dataset. The red line indicates the probability of recombination; substitutions, caused by either mutation or recombination, are represented as black crosses.

Visual inspection of the evolutionary events inferred by ClonalFrame in the divergence of the ST-8 complex indicated that recombination happened significantly more frequently than mutation, and that when it did it introduced a large number of substitutions, resulting in a much higher net effect of recombination than mutation in complex diversification (Fig. 3). The level of divergence of the imports, often above 1 %, demonstrated that they were likely to come from other meningococci, implying that the ST-8 complex was not sexually isolated from the rest of the species. Furthermore, a number of instances where only a fraction of a gene has been imported were observed, for example a fragment of the *fumC* gene in ST-66 (branch B, Fig. 3). Similar observations were made for the five other hyperinvasive lineages (data not shown).

### Lineage definition based on seven loci

**Fig. 4. f4:**
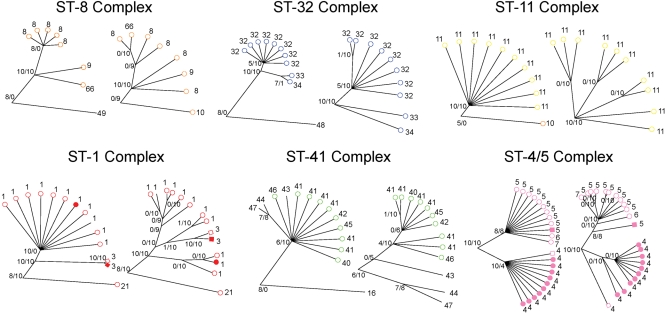
Comparison of the ClonalFrame analyses for the seven- and 20-locus datasets. For each of the six complexes, the seven-locus analyses are shown to the left and the 20-locus analyses to the right. The isolates are represented according to their MLEE designations as in Fig. 1, and labelled with their ST number according to the seven MLST gene fragments. The numbers attached to internal nodes of the ClonalFrame outputs are in the format *X*/*Y*, where *X* and *Y* are the confidence in the node according to the seven- and 20-locus analyses respectively. *X* and *Y* are expressed on a scale from 0 to 10.

Comparison of the ClonalFrame genealogies inferred from seven and 20 loci revealed a number of differences (Fig. 4). The 50 % majority-rule seven-locus consensus trees clustered isolates 297-0 (ST-49), BZ 147 (ST-48), B6116/77 (ST-10) and DK 24 (ST-16) in the ST-8, ST-32, ST-11 and ST-41 clonal complexes respectively (Fig. 4); however, all four assignments were not upheld if a more stringent rule was applied for the construction of the consensus tree, as indicated by their non-maximal support (8/10, 8/10, 5/10 and 8/10 respectively). In each case, the 20-locus analysis rejected the clusters (support of 0/10). MLEE designations provided further support for rejecting these clusters; for example, the MLEE designation of the isolate B6116/77 (ST-10) was cluster A4, whereas those of all isolates of the ST-11 complex were ET-37. The 20-locus analysis clustered this isolate within the ST-8 complex, with support 9/10, consistent with the fact that all isolates of the ST-8 complex were designated cluster A4 by MLEE. When a conservative 95 % cut-off value was employed, equivalent to keeping only the nodes with support equal to 10/10 in Fig. 4, the seven-locus analysis identified 7/8, 10/10, 10/10, 13/14 and 23/23 of the isolates from the ST-8, ST-32, ST-11, ST-1 and ST-4/5 complexes respectively. Only the definition of ST-41/44 complex was left unclear with a support of 6/10 for the 13 isolates. Inference based on the seven-locus dataset therefore had good power to define lineages, and a good accuracy, as no misassignments were made when using a 95 % confidence cut-off.

### Relationships within lineages

The seven-locus analysis defined four subgroups within the hyperinvasive lineages when a 95 % cut-off value was employed: three in the ST-1 complex and one in the ST-4/5 complex. Of these, two were confirmed by the 20-locus analysis, one was rejected and one remained unclear. The grouping that was rejected was the separation of the two ST-3 isolates from the ST-1 isolates. The one that remained unclear (support of 4/10 in the 20-locus analysis) was the monophyly of ST-4. A less conservative cut-off for the interpretation of the seven-locus results generated several misassignments. Furthermore, many relationships found with strong confidence by the 20-locus analysis were not inferred based on only seven loci, with 16 phylogenetic relationships not found at all (support of 0/10) and five found with weak statistical support (between 1/10 and 4/10). The seven-locus analysis therefore had low power to infer relationships within the six hyperinvasive lineages represented in this dataset.

**Fig. 5. f5:**
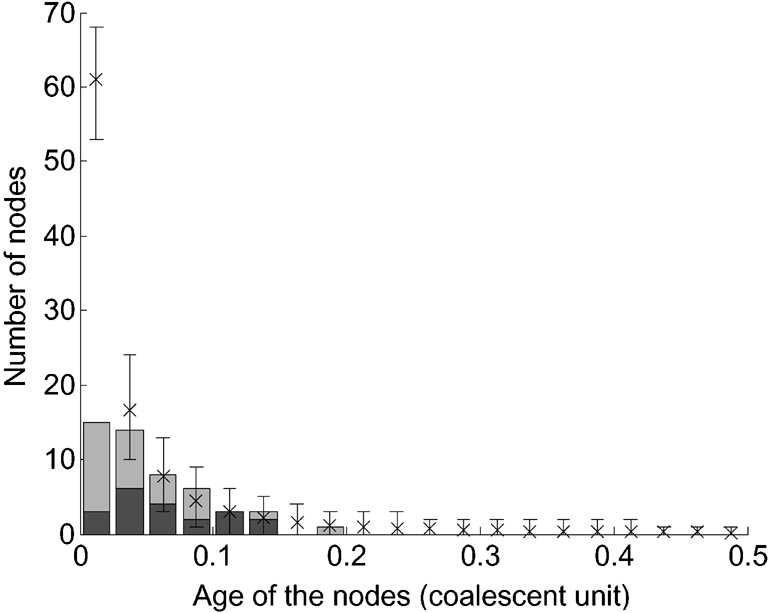
Number of nodes found compared to coalescent expectation. The numbers of clusters found by the 20-locus (light grey) and seven-locus (dark grey) analyses, sorted according to the inferred age of the clusters, are compared to the expectation (crosses) and 95 % credibility interval for the number of groups of different ages under a coalescent model computed using a Monte Carlo simulation (Kingman, 1982a, b).

The number of clusters found by the 20-locus analysis approximately followed the coalescent expectation (Kingman, 1982a, b), except for a deficit of recent as well as ancient clusters (Fig. 5). The missing recent clusters could be identified on the consensus tree (Fig. 4), where many branching orders were left unresolved for recently diverged clones, especially within the ST-32, ST-41 and ST-4 complexes, because of a lack of events distinguishing close relatives. The missing ancient clusters corresponded to an inability to reconstruct the branching order of the six hyperinvasive lineages among themselves and with the rest of the isolates, because the clonal signal was too disrupted (Fig. 1). When using only the seven MLST loci, there was sufficient statistical power to infer a large proportion of the expected clusters within only a narrow age range, centred approximately on 0.12 coalescent unit (Fig. 5), corresponding to the average estimated age of the six hyperinvasive lineages.

### Implications for epidemiological analyses

The utility of pathogen typing methods principally depends on the genealogical information they provide. Investigation of both disease outbreaks and longer-term epidemiological phenomena relies on distinguishing isolates that share a common ancestor from those that do not. For short-range epidemiology, typing methods are ideally highly discriminatory (Struelens, 1998), so that isolates that share a very recent common ancestor are grouped, while those from unrelated outbreaks are distinguishable. This requires that types evolve rapidly, which in turn entails that even very closely related isolates will occasionally have different types. In meningococci, for example, a number of approaches have been used, mostly based on variation in surface antigens and their genes (Jolley *et al.*, 2007) and are highly effective in outbreak detection and characterization (Elias *et al.*, 2006). Patterns of national or global spread, however, require the grouping of isolates that share more distant common ancestors (Caugant & Maiden, 2009). Thus, to be reliable and informative, typing methods need to be discriminatory, but also allow estimation of relationships between isolates that exhibit different types. DNA sequence data are particularly well suited for this, and stochastic models of sequence evolution have been developed to make such analysis possible, starting with the pioneering work of Jukes & Cantor (1969).

The unambiguous nature of MLST means that the spread of particular types can be tracked accurately, regardless of when or where the typing was performed (Maiden, 2006; Maiden *et al.*, 1998; Urwin & Maiden, 2003). Furthermore, sequence data taken from a variety of loci contain more information about genealogical relationships than most other data types, for example a single large sequenced region, although it poses the question how best to extract that information, given the confounding effect of recombination. Analyses of MLST data often assume that isolates sharing the same sequence type constitute an evolutionary unit. The extended dataset presented here shows that this assumption can be an oversimplification. The 20-locus analysis shows, for example, that grouping all isolates with MLST type ST-8 would be incorrect (Fig. 3). If only the data from the seven MLST loci are considered, ST-66 is a single locus variant of ST-8 (Fig. 3); however, the additional loci revealed several recombination and mutation events differentiating the ST-8 isolates from each other (lines A, D, I, J and G in Fig. 3). The recombination events in genes *dhps* and *gln* were also found in ST-66, and together provided strong evidence that the ST-8 strain above branch A was more closely related to ST-66 than it is to any of the other ST-8 strains in the sample (Fig. 3).

Closely related bacterial isolates contain few differences and thus relationships amongst them are difficult to resolve without the examination of a large number of potentially informative characters. On the other hand, deeper relationships are often obscured by extensive recombination (Holmes *et al.*, 1999). For this reason, intermediate branches in the genealogy will generally be the easiest to resolve. Depending on the topology of the tree, some branches will be intrinsically easier to resolve than others, whatever methods are used. This analysis shows that in *N. meningitidis*, the seven loci employed by routine MLST have substantial power to reconstruct clades in a narrow age range (Fig. 5). This age range corresponds to the age of the hyperinvasive lineages in *N. meningitidis*, and more generally to the concept of clonal complexes that has emerged from both MLEE and MLST studies (Caugant *et al.*, 1987; Urwin & Maiden, 2003). The clonal complexes are further characterized by gene content (Hotopp *et al.*, 2006), including the presence and absence of virulence factors (Bille *et al.*, 2008), antigenic properties, including capsules (Caugant & Maiden, 2009) and outer-membrane protein variants (Callaghan *et al.*, 2006; Urwin *et al.*, 2004), and the propensity to invade (Yazdankhah *et al.*, 2004). The addition of further loci provides limited improvements on the definition of these complexes (Fig. 4), which is expected given that the number of inferred groupings in this age range is in line with coalescent predictions (Fig. 5). The 20-locus analysis was, however, able to identify a large number of additional younger clades as well as some older clades (Fig. 5). This partially filled the gap in the overall distribution of ages for the reconstructed clades with that expected in a neutrally evolving population, although the youngest and oldest subdivisions remained unresolved (Fig. 5).

### Conclusions

These analyses demonstrate that the clonal complex concept captures an appreciable proportion of the information on genealogical relationships amongst *N. meningitidis* isolates available from the seven-locus MLST scheme. However, the seven-locus MLST data do not provide information on longer timescales, and the interrelationships among the lineages corresponding to clonal complexes remain unresolved, even with 20-locus data. This contrasts with the more extensive inferences that can be made from seven-locus MLST studies of some other genera, for example the *Bacillus cereus* group, where MLST data provide information on clonal relationships at multiple timescales (Didelot & Falush, 2007; Didelot *et al.*, 2009). These differences in the level of information contained in MLST datasets may due to variation in the homologous recombination rates across bacterial species (Feil *et al.*, 2001; Vos & Didelot, 2009).

The observation that clonal complexes exist in *N. meningitidis* is, in itself, consistent with neutral evolution and does not necessarily require any special non-neutral process such as the emergence of epidemic clones (Smith *et al.*, 1993); however, examination of the pattern of variation within and amongst complexes shows some evidence of deviations from neutrality, and several explanations have been invoked to accommodate them (Buckee *et al.*, 2008; Fraser *et al.*, 2005; Jolley *et al.*, 2005). The data presented here are consistent with such deviations (Fig. 2).

Finally, although seven-locus MLST and similar data are most powerful for resolving relationships at the level of clonal complex, many correlations of interest between genotype and phenotype occur at higher and at lower levels and this variation is a potentially rich source of biological inference. Variation in phenotype between closely related strains is particularly informative, since it occurs on a largely isogenic background (Falush & Bowden, 2006; Roumagnac *et al.*, 2006; Zhu *et al.*, 2001). Of particular interest is the ST-41/40 clonal complex, which is the most diverse of all meningococcal clonal complexes, representing 1895 of the 7606 STs (25 %) present in the *Neisseria* MLST database at the time of writing (July 2009) (Caugant & Maiden, 2009): members of this clonal complex exhibit diversity in their invasive phenotype, and will be particularly amenable to this type of analysis (Harrison *et al.*, 2009). Genomic data on the population scale, examining sufficient isolates that have carefully defined phenotypes, will allow relationships among meningococci to be resolved at multiple levels, allowing a richer description of epidemiological and evolutionary processes within bacterial populations (Maiden, 2008).

## Figures and Tables

**Table 1. t1:** Gene fragments analysed Annotations are from ENSEMBLE genomes (http://bacteria.ensembl.org) accessed 22 July 2009, except where a different locus name is customarily used in the MLST scheme. In these cases the ENSEMBLE designation is given in parentheses.

**Locus**	**Annotated function**	**Fragment length (bp)**	**Polymorphic sites (%)**	**No. of alleles**	***d*_N_/*d*_S_**
*abcZ*	Putative ABC transporter ATP-binding protein	433	17.3	15*	0.049
*adk*	Adenylate kinase	465	3.7	10*	0.010
*aroE*	Shikimate dehydrogenase	490	33.9	19*	1.545
*fumC*	Fumarate hydratase class II	465	8.2	19*	0.022
*gdh*	Glucose-6-phosphate 1-dehydrogenase (*zwf*)	501	5.6	16*	0.045
*mtg*	Monofunctional biosynthetic peptidoglycan transglycosylase (*mtgA*)	497	15.3	16*	0.115
*pdhC*	Pyruvate dehydrogenase subunit E1 (*aceE*)	480	16.7	24*	0.063
*pgm*	Phosphoglucomutase	450	17.1	21*	0.119
*pilA*	Probable signal recognition particle protein	432	11.6	36*	0.002
*pip*	Proline iminopeptidase	416	6.2	19*	0.449
*ppk*	Polyphosphate kinase	579	13.3	23*	0.088
*serC*	Phosphoserine aminotransferase	451	14.9	29*	0.123
*aspA*	Aspartate ammonia-lyase	432	6.7	11†	0.078
*carB*	Carbamoyl phosphate synthase large subunit	495	9.7	15†	0.014
*dhps*	Dihydropteroate synthase	366	21.2	33†	0.189
*glnA*	Glutamine synthetase	471	13.0	11†	0.036
*rpiA*	Ribose-5-phosphate isomerase A	567	8.2	21†	0.032
*tal*	Transaldolase	492	11.0	15†	0.237
*gpm*	2,3-Bisphosphoglycerate-dependent phosphoglycerate mutase (*gpmA*)	534	5.0	18†	0.163
*pyk*	Pyruvate kinase (*pykA*)	453	10.4	17†	0.071

*From 107 isolates. †From 93 isolates.

**Table 2. t2:** Properties of the six hyperinvasive lineages

	**Clonal complex**					**ST-4/5**
	**ST-8**	**ST-32**	**ST-11**	**ST-1**	**ST-41/44**	
**Content of complexes**						
No. of isolates	8	10	10	14	13	23
No. of ETs*	1	1	1	5	2	4
No. of MLST STs	4	3	1	3	8	4
No. of types (all fragments)	8	8	8	14	12	14
**Descriptive statistics (based on 20 genes dataset)**						
No. of polymorphic sites	270	162	121	395	314	246
No. of polymorphic fragments	12	9	9	16	17	14
Mean pairwise distance	0.009	0.004	0.004	0.010	0.008†	0.007
**ClonalFrame analysis**						
Relative age‡	0.3 [0.1;0.5]	0.2 [0.1;0.4]	0.1 [0.1;0.2]	0.4 [0.2;0.6]	0.3 [0.2;0.5]§	0.2 [0.1;0.4]
No. of mutation events||	1.6 [0.6;3.3]	0.5 [0.2;1.5]	0.9 [0.6;1.6]	8.8 [5.5;13.2]	5.8 [3.1;9.8]	0.9 [0.2;1.8]
No. of recombination events||	5.1 [3.6;7.1]	3.0 [2.0;5.1]	1.5 [1.2;2.2]	12.9 [10.6;15.4]	9.5 [6.0;14.4]	5.4 [4.7;6.1]
No. of substitutions via recombination||	85 [65;107]	49 [28;76]	22 [19;36]	121 [94;138]	83 [53;120]	58 [56;63]
Proportion of clonal frame||	80 % [74;86]	87 % [83;91]	92 % [90;93]	72 % [68;79]	75 % [69;82]	81 % [79;82]
*ρ*/θ	3.8 [1.5;9.2]	6.8 [2.0;14.5]	1.7 [0.8;3.5]	1.5 [0.9;2.6]	1.7 [0.9;2.9]	7.7 [2.7;26.5]
*r*/*m*	63.7 [25.5;144.6]	108.9 [35.7;217.6]	25.7 [13.8;45.6]	14.4 [8.7;23.0]	15.2 [8.5;24.6]	82.3 [31.2;271.9]
External/internal branch length ratio	3.9 [1.7;7.3]	2.3 [0.8;4.3]	2.4 [0.9;5.1]	1.4 [1.1;1.9]	2.8 [1.4;5.1]	1.9 [1.0;3.0]
Statistical significance¶	0.012	0.047	0.046	0.045	0.006	0.004

*Electrophoretic types as determined by MLEE. †Between the isolates of lineage 3 and the three others, the mean per site pairwise distance is 0.015. ‡As a fraction of the estimated age of *N. meningitidis.* The numbers in square brackets in this and subsequent rows are 95% credibility intervals. §The relative age of the group of isolates of lineage 3 is 0.2 [0.1;0.3]. ||Measured between each isolate of each complex and their most recent common ancestor. ¶Deviation from random expectation under the Kingman coalescent (Kingman, 1982a).

## Abbreviations

- MLST, multilocus sequence typing
- MLEE, multilocus enzyme electrophoresis
- ST, sequence type

## References

[r1] Achtman, M. (1994). Clonal spread of serogroup A meningococci. A paradigm for the analysis of microevolution in bacteria. Mol Microbiol 11, 15–22. 8145638 10.1111/j.1365-2958.1994.tb00285.x

[r2] Achtman, M. (1996). A surfeit of YATMs? J Clin Microbiol 34, 1870 8964891 10.1128/jcm.34.7.1870-1870.1996 PMC229142

[r3] Bentley, S. D., Vernikos, G. S., Snyder, L. A., Churcher, C., Arrowsmith, C., Chillingworth, T., Cronin, A., Davis, P. H., Holroyd, N. E. & other authors (2007). Meningococcal genetic variation mechanisms viewed through comparative analysis of serogroup C strain FAM18. PLoS Genet 3, e23 17305430 10.1371/journal.pgen.0030023 PMC1797815

[r4] Bille, E., Zahar, J. R., Perrin, A., Morelle, S., Kriz, P., Jolley, K. A., Maiden, M. C., Dervin, C., Nassif, X. & other authors (2005). A chromosomally integrated bacteriophage in invasive meningococci. J Exp Med 201, 1905–1913. 15967821 10.1084/jem.20050112 PMC2212043

[r5] Bille, E., Ure, R., Gray, S. J., Kaczmarski, E. B., McCarthy, N. D., Nassif, X., Maiden, M. C. & Tinsley, C. R. (2008). Association of a bacteriophage with meningococcal disease in young adults. PLoS One 3, e3885 19065260 10.1371/journal.pone.0003885 PMC2587699

[r6] Brooks, S. P. & Gelman, A. (1998). General methods for monitoring convergence of iterative simulations. J Comput Graph Statist 7, 434–455.

[r7] Buckee, C. O., Jolley, K., Recker, M., Penman, B., Kriz, P., Gupta, S. & Maiden, M. C. (2008). Role of selection in the emergence of lineages and the evolution of virulence in *Neisseria meningitidis*. Proc Natl Acad Sci U S A 105, 15082–15087. 18815379 10.1073/pnas.0712019105 PMC2553036

[r8] Bygraves, J. A., Urwin, R., Fox, A. J., Gray, S. J., Russell, J. E., Feavers, I. M. & Maiden, M. C. J. (1999). Population genetic and evolutionary approaches to the analysis of *Neisseria meningitidis* isolates belonging to the ET-5 complex. J Bacteriol 181, 5551–5556. 10482493 10.1128/jb.181.18.5551-5556.1999 PMC94072

[r9] Callaghan, M. J., Jolley, K. A. & Maiden, M. C. (2006). Opacity-associated adhesin repertoire in hyperinvasive *Neisseria meningitidis*. Infect Immun 74, 5085–5094. 16926400 10.1128/IAI.00293-06 PMC1594835

[r10] Caugant, D. A. (2008). Genetics and evolution of *Neisseria meningitidis*: importance for the epidemiology of meningococcal disease. Infect Genet Evol 8, 558–565. 18479979 10.1016/j.meegid.2008.04.002

[r11] Caugant, D. A. & Maiden, M. C. (2009). Meningococcal carriage and disease – population biology and evolution. Vaccine 27 (*Suppl. 2*), B64–B70. 19464092 10.1016/j.vaccine.2009.04.061 PMC2719693

[r12] Caugant, D. A., Mocca, L. F., Frasch, C. E., Frøholm, L. O., Zollinger, W. D. & Selander, R. K. (1987). Genetic structure of *Neisseria meningitidis* populations in relation to serogroup, serotype, and outer membrane protein pattern. J Bacteriol 169, 2781–2792. 3108242 10.1128/jb.169.6.2781-2792.1987 PMC212185

[r13] Didelot, X. & Falush, D. (2007). Inference of bacterial microevolution using multilocus sequence data. Genetics 175, 1251–1266. 17151252 10.1534/genetics.106.063305 PMC1840087

[r14] Didelot, X., Barker, M., Falush, D. & Priest, F. G. (2009). Evolution of pathogenicity in the *Bacillus cereus* group. Syst Appl Microbiol 32, 81–90. 19200684 10.1016/j.syapm.2009.01.001

[r15] Elias, J., Harmsen, D., Claus, H., Hellenbrand, W., Frosch, M. & Vogel, U. (2006). Spatiotemporal analysis of invasive meningococcal disease, Germany. Emerg Infect Dis 12, 1689–1695. 17283618 10.3201/eid1211.060682 PMC3372358

[r16] Falush, D. & Bowden, R. (2006). Genome-wide association mapping in bacteria? Trends Microbiol 14, 353–355. 16782339 10.1016/j.tim.2006.06.003

[r17] Feavers, I. M., Gray, S. J., Urwin, R., Russell, J. E., Bygraves, J. A., Kaczmarski, E. B. & Maiden, M. C. J. (1999). Multilocus sequence typing and antigen gene sequencing in the investigation of a meningococcal disease outbreak. J Clin Microbiol 37, 3883–3887. 10565901 10.1128/jcm.37.12.3883-3887.1999 PMC85836

[r18] Feil, E. J., Maiden, M. C. J., Achtman, M. & Spratt, B. G. (1999). The relative contributions of recombination and mutation to the divergence of clones of *Neisseria meningitidis*. Mol Biol Evol 16, 1496–1502. 10555280 10.1093/oxfordjournals.molbev.a026061

[r19] Feil, E. J., Holmes, E. C., Bessen, D. E., Chan, M. S., Day, N. P., Enright, M. C., Goldstein, R., Hood, D. W., Kalia, A. & other authors (2001). Recombination within natural populations of pathogenic bacteria: short-term empirical estimates and long-term phylogenetic consequences. Proc Natl Acad Sci U S A 98, 182–187. 11136255 10.1073/pnas.98.1.182 PMC14565

[r20] Fiala, K. L. & Sokal, R. R. (1985). Factors determining the accuracy of cladogram estimation – evaluation using computer-simulation. Evolution 39, 609–622. 28561973 10.1111/j.1558-5646.1985.tb00398.x

[r21] Fraser, C., Hanage, W. P. & Spratt, B. G. (2005). Neutral microepidemic evolution of bacterial pathogens. Proc Natl Acad Sci U S A 102, 1968–1973. 15684071 10.1073/pnas.0406993102 PMC548543

[r22] Gelman, A. (1996). Inference and monitoring convergence. In *Markov Chain Monte Carlo in Practice*. Edited by W. R. Gilks, S. Richardson & D. Spiegelhalter. Boca Raton, FL: Chapman & Hall.

[r23] Gelman, A. & Rubin, D. B. (1992). Inference from iterative simulation using mulitple sequences. Stat Sci 7, 457–472.

[r24] Guttman, D. S. (1997). Recombination and clonality in natural populations of *Escherichia coli*. Trends Ecol Evol 12, 16–22. 21237956 10.1016/s0169-5347(96)10057-4

[r25] Guttman, D. S. & Dykhuizen, D. E. (1994). Clonal divergence in *Escherichia coli* as a result of recombination, not mutation. Science 266, 1380–1383. 7973728 10.1126/science.7973728

[r26] Harrison, O. B., Evans, N. J., Blair, J. M., Grimes, H. S., Tinsley, C. R., Nassif, X., Kriz, P., Ure, R., Gray, S. J. & other authors (2009). Epidemiological evidence for the role of the hemoglobin receptor, HmbR, in meningococcal virulence. J Infect Dis 200, 94–98. 19476432 10.1086/599377 PMC2731217

[r27] Holmes, E. C., Urwin, R. & Maiden, M. C. J. (1999). The influence of recombination on the population structure and evolution of the human pathogen *Neisseria meningitidis*. Mol Biol Evol 16, 741–749. 10368953 10.1093/oxfordjournals.molbev.a026159

[r28] Hotopp, J. C., Grifantini, R., Kumar, N., Tzeng, Y. L., Fouts, D., Frigimelica, E., Draghi, M., Giuliani, M. M., Rappuoli, R. & other authors (2006). Comparative genomics of *Neisseria meningitidis*: core genome, islands of horizontal transfer and pathogen-specific genes. Microbiology 152, 3733–3749. 17159225 10.1099/mic.0.29261-0

[r29] Jodar, L., Feavers, I. M., Salisbury, D. & Granoff, D. M. (2002). Development of vaccines against meningococcal disease. Lancet 359, 1499–1508. 11988262 10.1016/S0140-6736(02)08416-7

[r30] Jolley, K. A., Kalmusova, J., Feil, E. J., Gupta, S., Musilek, M., Kriz, P. & Maiden, M. C. (2000). Carried meningococci in the Czech Republic: a diverse recombining population. J Clin Microbiol 38, 4492–4498. 11101585 10.1128/jcm.38.12.4492-4498.2000 PMC87626

[r31] Jolley, K. A., Chan, M. S. & Maiden, M. C. (2004). mlstdbNet – distributed multi-locus sequence typing (MLST) databases. BMC Bioinformatics 5, 86 15230973 10.1186/1471-2105-5-86 PMC459212

[r32] Jolley, K. A., Wilson, D. J., Kriz, P., McVean, G. & Maiden, M. C. (2005). The influence of mutation, recombination, population history, and selection on patterns of genetic diversity in *Neisseria meningitidis*. Mol Biol Evol 22, 562–569. 15537808 10.1093/molbev/msi041

[r33] Jolley, K. A., Brehony, C. & Maiden, M. C. (2007). Molecular typing of meningococci: recommendations for target choice and nomenclature. FEMS Microbiol Rev 31, 89–96. 17168996 10.1111/j.1574-6976.2006.00057.x

[r34] Jukes, T. H. & Cantor, C. R. (1969). Evolution of protein molecules. In *Mammalian Protein Metabolism*, pp. 21–132. Edited by H. N. Munro. New York: Academic Press.

[r35] Kingman, J. F. (1982a). The coalescent. Stochastic Process Appl 13, 235–248.

[r36] Kingman, J. F. C. (1982b). On the genealogy of large populations. J Appl Probab 19, 27–43.

[r37] Kristiansen, B.-E., Radstrom, P., Jenkins, A., Ask, E., Facinelli, B. & Sköld, O. (1990). Cloning and characteriztion of a DNA fragment that confers sulfonamide resistance in a serogroup B, serotype 15 strain of *Neisseria meningitidis*. Antimicrob Agents Chemother 34, 2277–2279. 2127350 10.1128/aac.34.11.2277 PMC172039

[r38] Levin, B. R. (1981). Periodic selection, infectious gene exchange and the genetic structure of *E. coli* populations. Genetics 99, 1–23. 7042453 10.1093/genetics/99.1.1 PMC1214481

[r39] Linz, B., Schenker, M., Zhu, P. & Achtman, M. (2000). Frequent interspecific genetic exchange between commensal Neisseriae and *Neisseria meningitidis*. Mol Microbiol 36, 1049–1058. 10844690 10.1046/j.1365-2958.2000.01932.x

[r40] Maiden, M. C. (2006). Multilocus sequence typing of bacteria. Annu Rev Microbiol 60, 561–588. 16774461 10.1146/annurev.micro.59.030804.121325

[r41] Maiden, M. C. (2008). Population genomics: diversity and virulence in the *Neisseria*. Curr Opin Microbiol 11, 467–471. 18822386 10.1016/j.mib.2008.09.002 PMC2612085

[r42] Maiden, M. C. J. & Spratt, B. G. (1999). Meningococcal conjugate vaccines: new opportunities and new challenges. Lancet 354, 615–616. 10466659 10.1016/s0140-6736(99)00252-4

[r43] Maiden, M. C. J., Bygraves, J. A., Feil, E., Morelli, G., Russell, J. E., Urwin, R., Zhang, Q., Zhou, J., Zurth, K. & other authors (1998). Multilocus sequence typing: a portable approach to the identification of clones within populations of pathogenic microorganisms. Proc Natl Acad Sci U S A 95, 3140–3145. 9501229 10.1073/pnas.95.6.3140 PMC19708

[r44] Milkman, R. & Bridges, M. M. (1990). Molecular evolution of the *Escherichia coli* chromosome. III. Clonal frames. Genetics 126, 505–517. 1979037 10.1093/genetics/126.3.505 PMC1204208

[r45] Musser, J. M. (1996). Molecular population genetic analysis of emerged bacterial pathogens: selected insights. Emerg Infect Dis 2, 1–17. 8903193 10.3201/eid0201.960101 PMC2639800

[r46] Roumagnac, P., Weill, F. X., Dolecek, C., Baker, S., Brisse, S., Chinh, N. T., Le, T. A., Acosta, C. J., Farrar, J. & other authors (2006). Evolutionary history of *Salmonella typhi*. Science 314, 1301–1304. 17124322 10.1126/science.1134933 PMC2652035

[r47] Schierup, M. H. & Hein, J. (2000). Consequences of recombination on traditional phylogenetic analysis. Genetics 156, 879–891. 11014833 10.1093/genetics/156.2.879 PMC1461297

[r48] Smith, M. J., Smith, N. H., O'Rourke, M. & Spratt, B. G. (1993). How clonal are bacteria? Proc Natl Acad Sci U S A 90, 4384–4388. 8506277 10.1073/pnas.90.10.4384 PMC46515

[r49] Staden, R. (1996). The Staden sequence analysis package. Mol Biotechnol 5, 233–241. 8837029 10.1007/BF02900361

[r50] Stephens, D. S. (2009). Biology and pathogenesis of the evolutionarily successful, obligate human bacterium *Neisseria meningitidis*. Vaccine 27 (*Suppl. 2*), B71–B77. 19477055 10.1016/j.vaccine.2009.04.070 PMC2712446

[r51] Struelens, M. J. (1998). Molecular epidemiologic typing systems of bacterial pathogens: current issues and perspectives. Mem Inst Oswaldo Cruz 93, 581–585. 9830521 10.1590/s0074-02761998000500004

[r52] Tondella, M. L., Reeves, M. W., Popovic, T., Rosenstein, N., Holloway, B. P. & Mayer, L. W. (1999). Cleavase fragment length polymorphism analysis of *Neisseria meningitidis* basic metabolic genes. J Clin Microbiol 37, 2402–2407. 10405375 10.1128/jcm.37.8.2402-2407.1999 PMC85239

[r53] Urwin, R. & Maiden, M. C. (2003). Multi-locus sequence typing: a tool for global epidemiology. Trends Microbiol 11, 479–487. 14557031 10.1016/j.tim.2003.08.006

[r54] Urwin, R., Russell, J. E., Thompson, E. A., Holmes, E. C., Feavers, I. M. & Maiden, M. C. (2004). Distribution of surface protein variants among hyperinvasive meningococci: implications for vaccine design. Infect Immun 72, 5955–5962. 15385499 10.1128/IAI.72.10.5955-5962.2004 PMC517544

[r55] van Belkum, A., Struelens, M., de Visser, A., Verbrugh, H. & Tibayrenc, M. (2001). Role of genomic typing in taxonomy, evolutionary genetics, and microbial epidemiology. Clin Microbiol Rev 14, 547–560. 11432813 10.1128/CMR.14.3.547-560.2001 PMC88989

[r56] Vos, M. & Didelot, X. (2009). A comparison of homologous recombination rates in bacteria and archaea. ISME J 3, 199–208. 18830278 10.1038/ismej.2008.93

[r57] Wang, J.-F., Caugant, D. A., Li, X., Hu, X., Poolman, J. T., Crowe, B. A. & Achtman, M. (1992). Clonal and antigenic analysis of serogroup A *Neisseria meningitidis* with particular reference to epidemiological features of epidemic meningitis in China. Infect Immun 60, 5267–5282. 1452360 10.1128/iai.60.12.5267-5282.1992 PMC258306

[r58] Womble, D. D. (2000). GCG: the Wisconsin Package of sequence analysis programs. Methods Mol Biol 132, 3–22. 10547828 10.1385/1-59259-192-2:3

[r59] Yazdankhah, S. P., Kriz, P., Tzanakaki, G., Kremastinou, J., Kalmusova, J., Musilek, M., Alvestad, T., Jolley, K. A., Wilson, D. J. & other authors (2004). Distribution of serogroups and genotypes among disease-associated and carried isolates of *Neisseria meningitidis* from the Czech Republic, Greece, and Norway. J Clin Microbiol 42, 5146–5153. 15528708 10.1128/JCM.42.11.5146-5153.2004 PMC525265

[r60] Zhu, P., van der Ende, A., Falush, D., Brieske, N., Morelli, G., Linz, B., Popovic, T., Schuurman, I. G., Adegbola, R. A. & other authors (2001). Fit genotypes and escape variants of subgroup III *Neisseria meningitidis* during three pandemics of epidemic meningitis. Proc Natl Acad Sci U S A 98, 5234–5239. 11287631 10.1073/pnas.061386098 PMC33193

